# An age-matched comparative study of ocular biometry parameters in cataractous and non-cataractous eyes of children in Ibadan, Nigeria

**DOI:** 10.4314/gmj.v58i4.5

**Published:** 2024-12

**Authors:** Ezinne O Onebunne, Mary O Ugalahi, Bolutife A Olusanya, Charles O Bekibele

**Affiliations:** 1 Department of Ophthalmology, University College Hospital Ibadan, Nigeria; 2 Department of Ophthalmology, University College Hospital/College of Medicine, University of Ibadan Nigeria

**Keywords:** Cataract, biometry, Nigerian, Child, Control group

## Abstract

**Objective:**

This study compared the preoperative ocular biometric parameters of children who had cataract with age-matched healthy children to provide an understanding of a range of typical measurements in indigenous African eyes and aid management of childhood cataract.

**Design:**

Observational, cross-sectional, comparative study of two groups- cases and controls.

**Setting:**

Child eye health tertiary health facility

**Participants:**

Group A consisted of children aged 2 to 6 years with cataracts, and Group B of age-matched controls. Ocular biometry measurements including keratometry, axial length (AXL), pachymetry (central corneal thickness [CCT]) and tonometry were measured.

**Results:**

Thirty eyes of 24 children were studied in each group. Seventeen (70.8%) children had bilateral cataracts. The mean age of the cataract group (A) was 5.97±2.93 years, while that of the control group (B) was 6.33±2.89 years. The mean preoperative values for the ocular biometric parameters were: Group A AXL= 23.4(±1.4) mm versus Group B AXL= 22.7(±0.9) mm, p= 0.028; Group A CCT= 563.6(±59.7) µm versus Group B CCT= 551.0(±36.4) µm, p= 0.33; and Group A K = 43.4(±2.3) D versus Group B K = 42.5(±1.5) D, p= 0.08. Group A IOP was 13.7(±2.8) mmHg, while Group B IOP was 13.8 (±3.1) mmHg, p =0.93.

**Conclusion:**

There was no statistically significant difference in ocular biometric parameters between children's eyes with and without cataracts except for the axial length.

**Funding:**

None declared

## Introduction

In Sub-Saharan Africa, cataracts are a significant cause of childhood blindness.^1^ Its prevalence in lower-middleincome economies is 0.32-8.49 per 10,000.^2^ Cataract surgery is the most commonly performed intraocular surgery in the pediatric population.^3^ However, managing pediatric cataracts is challenging, both pre-operatively, intra-operatively, and post-operatively. Each of these stages plays a significant role in the amblyogenic potential of cataracts.

Axial length (AXL) and keratometry play a significant role in the intraocular lens (IOL) calculation formula. Zhan and colleagues^4^ elucidated refocusing and form deprivation as proposed mechanisms underlying the changes in AXL in patients with congenital cataracts. They outlined some factors that induce variations in the ocular axis of children with congenital cataracts, such as the preoperative onset of form deprivation, surgery date, degree of amblyopia postoperatively, IOL implantation and the existence of normal stereopsis and fusion. They established that a consensus is yet to be reached on AXL growth and development patterns in children with congenital cataracts.

Glaucoma is a serious complication following pediatric cataract surgery; thus, appropriate criteria for diagnosis need careful deliberation. Intraocular pressure (IOP) values with applanation tonometry tend to be overestimated and underestimated in eyes with thick and thin corneas, respectively.^5^ Variables that affect IOP, such as CCT, affect treatment and may even lead to a misdiagnosis in the pediatric population.^6^

Investigators have found longer AXL, thinner CCT, and flatter mean keratometry in African American eyes compared with Caucasians.^7^–^11^

Nihalani et al.^12^ found a trend toward longer AXL and steeper K and increasing variability in preoperative biometry measures in eyes with pediatric cataracts compared to age-matched controls. None of these studies have been carried out in Indigenous black populations yet.

Understanding deviations in ocular biometric parameters from normative data could be valuable in mitigating the refractive surprises observed in eyes with pediatric cataracts.^12^ Additionally, understanding a range of typical measurements could assist surgeons in recognising atypical parameters and may indicate a need for re-assessment of measurement accuracy. Studies in developed countries have developed algorithms based on ocular biometrics for accurate IOL selection.^8^ How Indigenous African eyes compare to these growth curves remains unknown, as there is a gap in the literature specifically reporting biometry data for pediatric eyes in Indigenous African populations. Thus, this study directly compares the preoperative ocular biometrics in children with cataracts with age-matched controls in an Indigenous African population.

## Methods

This was a hospital-based, double-armed cohort study involving patients who had pediatric cataracts and age-matched controls at the University College Hospital (UCH), Ibadan, a child eye health tertiary health facility in Southwestern Nigeria, between December 2020 and December 2021. After ethical approval was obtained from the University College Hospital / University of Ibadan (UCH/UI) Ethics Committee (UI/EC/20/0222), all patients and parents received a thorough explanation of the study design and aims. Thereafter, written informed consent was obtained from each parent, while assent was obtained from older patients before enrollment. Study participants in group A were children aged 2 to 16 years at their preoperative visit for cataract surgery. Patients were excluded if they had traumatic or uveitic cataracts, microcornea, microphthalmia, glaucoma, keratoconus, severe VKC, aniridia, cornea scar, ectopia lentis, previous ophthalmic surgical intervention, cerebral palsy, or Down syndrome. Children under 2 years old were excluded as their biometric values undergo rapid changes at that age. The control group were age-matched children with normal eye examination or/mild refractive error less than ±1.00D. These were children recruited from the eye clinic with a normal eye examination or mild refractive error. Matching was done by age category using age ± 1 year.

One investigator conducted all clinical examinations, including autorefraction, slit lamp examination, tonometry, and ocular biometric examinations, including A-scan biometry, keratometry, and pachymetry. Visual acuity was obtained using the Snellen chart. In preverbal children, matching optotype tests (the Leas Optotype and Kay Picture chart) were used. Anterior segment examination was performed using a slit lamp biomicroscope (a portable, handheld type was used where necessary, especially in children examined under sedation). A diagnosis of cataract was confirmed following a slit lamp examination.

Intraocular pressures were measured using Perkins handheld tonometer for the younger children and the Goldman applanation tonometer for the older child. At least 2 measurements were obtained for each eye and the average value was recorded. Keratometry was performed using a KR-8900 autorefractor-keratometer (Topcon Corporation, Tokyo, Japan). Automated measurements of the cornea curvature were made using optical sensors. The steepest and flattest values were displayed numerically, and the average value was included in the study. Axial length and pachymetry values were obtained using the Appascan MAX AP 0518-AP-064 ultrasonic biometer (Appasamy associates Chennai-600106, INDIA). The children's eyes were anaesthetised with one drop of 0.5% tetracaine before the procedure. The average of five axial length and pachymetry measurements was taken and accepted only if these five readings' standard deviation (SD) was less than 0.05 mm for AXL and 5µm for pachymetry. If not, the measurements were repeated till the best five acceptable measurements were obtained. Some of the younger patients who were unable to cooperate actively were sedated using chloral hydrate (80-100mg/kg, maximum dose 1g, oral administration).

A minimum sample size of 23 patients per group was calculated using the sample size formula to assess the difference in mean values of a quantitative variable between cases and controls with 1:1 control to a case.^13^ A standard normal variate corresponding to the significance level at 5% type 1 error and a power of 80% was used. The standard deviation was from a previous study.^14^

Demographic data and clinical characteristics were summarised using frequency counts, mean, and standard deviation. The independent t-test and one-way ANOVA were used to explore the difference in mean values of the ocular parameters. Levene's test of homogeneity of variances was used to explore the differences in variance.

Pearson's correlation analysis was also used to evaluate the relationships between biometry parameters in the ocular parameters (AXL, CCT, K) and clinical and demographic characteristics. A p-value less than 0.05 was statistically significant.

The data was analysed using IBM Statistical Package for the Social Sciences (IBM SPSS) for Windows software version 22 (SPSS Inc., Chicago, IL, USA).

## Results

The cataractous eyes of patients with unilateral cataracts and unoperated eyes of children with bilateral cataracts were recruited, as some patients with bilateral cataracts had surgery performed in the contralateral eye prior to recruitment. There were 30 eyes of 24 children with cataracts and 30 eyes of 24 age-matched controls. The mean age in the cataract group (5.97 ± 2.93 years) was comparable to the control group (6.33 ± 2.90 years), p = 0.63. Both groups were comparable in their anthropometric characteristics, such as a height of 120.26 ±17.09cm in the cataract group versus 120.19 ± 18.63 cm (p = 0.99) in the control group. Also, a similar mean weight was obtained in the cataract group, 21.26 ±5.4 kg and in the control group, 22.08 ± 8.6kg (p = 0.71).

The mean BMI in the cataract group was 14.42 ± 2.03 kg/m2 and 14.81 ± 1.90 kg/m^2^ (p = 0.89). In the control group, there was an almost equal male (53.3%) to female (46.7%) ratio of 1.1:1. In the group with cataract, there were 18 (75%) males with a male/female ratio of 3:1. Males were significantly younger than female children in the group with cataract (Table 1).

**Table 1:** Distribution of children with cataracts by sociodemographic and clinical characteristics

	Frequency (%)	Mean age (SD)	p-value*
Gender (n = 24 patients)			
Male	18(75.0)	4.96(2.51)	<0.001
Female	6(25.0)	9.29(1.25)	
Cataract laterality (n = 24 patients)			
Bilateral	17(70.8)	5.48(2.41)	0.19
Unilateral	7(29.2)	7.71(3.95)	
Cataract aetiology (n = 30 eyes)			
Congenital	14(46.7)	5.36(2.79)	0.29
Developmental	16(53.3)	6.50(3.03)	
Cataract morphology (n = 30 eyes)			
Full	12(40.0)		
Lamella	13(43.3)		
Posterior polar	2(6.7)		
Lamella + anterior polar	1(3.3)		
Lamella + posterior polar	1(3.3)		
Nuclear	1(3.3)		
Preoperative Visual acuity (n = 30 eyes)	1(3.3)		
<6/12 – 6/18	3(9.9)		
<6/18 – 6/60	23(76.7)		
<3/60	3(9.9)		
LP			

* - p-value from independent T-test, LP – Light perception, SD – standard deviation

There was no significant difference in age distribution based on cataract laterality or aetiology. About half of the patients had lamellar cataracts. In eyes with cataracts, visual acuity ranged from light perception to 6/18. Both the uncorrected and best-corrected visual acuity was 6/6 in all the controls. The mean values of the ocular biometric parameters, including IOP, are presented below for cases and controls (Table 2).

**Table 2:** Comparison of AXL, CCT, K and IOP measurement values between cases and their controls preoperatively

Ocular biometric Parameter	Patients	Control	p-value
**Axial length (mm)**	23.4(±1.4)	22.7(±0.9)	0.028
**Range**	19.5 - 27.5	21.1 - 24.5	
**Central Corneal Thickness (µm)**	563.6(±59.7)	551.0(±36.4)	0.33
**Range**	487.5 - 753.0	475.0 - 612.5	
**Keratometry (D)**	43.4(±2.3)	42.5(±1.5)	0.08
**Range**	38.8 - 48.5	39.8 - 45.8	
**Intraocular pressure (mmHg)**	13.71(±2.78)	13.79(±3.13)	0.927
**Range**	10.00 – 20.00	10.00 – 20.00	

AXL - Axial length, CCT - Central corneal thickness, IOP - Intraocular pressure, SD - Standard deviation

The ocular biometric parameters in the cataract group were explored based on gender, laterality of cataract and aetiology of cataract, but no statistically significant differences were found (Table 3). The ocular biometric parameters in the control group were explored based on gender and age (Table 4). We found thinner central cornea in females. Mean AXL and keratometry values differed significantly across the age categories.

**Table 3:** Comparison of mean AXL, CCT, K and IOP values in cases by gender, age, aetiology and laterality of cataract

Parameter	AXL (mm)		CCT (µm)		K (D)		IOP (mmHg)	
	Mean (SD)	p-value	Mean (SD)	p-value	Mean (SD)	p-value	Mean (SD)	p-value
**Overall**	23.4 (±1.4)		563.6 (±59.7)		43.4 (±2.3)		13.7 (±2.8)	
**Gender**								
**Male**	23.5 (±1.5)	0.29	571.3 (±63.6)	0.24	43.2 (±2.2)	0.33	14.1 (±2.8)	0.32
**Female**	22.9 (±0.5)		538.1 (±37.5)		44.1 (±2.4)		12.6 (±2.6)	
Age category**								
**2-4 years (12 eyes)**	23.0 (±1.4)	0.11	551.0 (±40.3)		43.7 (±2.8)		14.3 (±2.6)	
**5-8 years (11 eyes)**	24.0 (±1.5)		580.9 (±83.3)		43.7 (±2.0)			
**9-13 years (7 eyes)**	22.9 (±0.5)		557.1 (±36.4)	0.52	42.5 (±1.6)	0.52	12.0 (±2.8)	0.12
**Laterality of cataract**								
**Bilateral**	23.4 (±1.5)	0.75	568.9 (±63.3)	0.37	43.6 (±2.5)	0.29	14.1 (±2.2)	0.33
**Unilateral**	23.2 (±0.4)		541.7 (±29.1)		42.5 (±1.3)		12.9 (±3.8)	
**Aetiology of cataract**								
**Congenital**	23.0 (±1.4)	0.21	575.1 (±71.1)	0.30	44.3 (±2.9)	0.05	13.5 (±2.1)	0.83
**Developmental**	23.7 (±1.3)		550.3 (±41.9)		42.6 (±1.2)		13.8 (±3.1)	

AXL - axial length, CCT - central corneal thickness, K – keratometry, IOP - intraocular pressure

** - p value from ANOVA

**Table 4:** Comparison of mean AXL, CCT, K and IOP values in controls by gender and age

Parameter	AXL (mm)		CCT (µm)		K (D)		IOP (mmHg)	
	Mean (SD)	p-value	Mean (SD)	p-value	Mean (SD)	p-value	Mean (SD)	p-value
**Overall Means**	22.7±0.9		551.0±36.4		42.5±1.5		13.8±3.1	
**Means by Gender**								
**Male**	22.8 (±0.9)		567.1 (±40.6)		42.4 (±1.3)		13.3 (±2.9)	
**Female**	22.6 (±1.0)	0.54	532.5 (±19.2)	0.006	2.7 (±1.9)	0.61	14.4 (±3.4)	0.37
Means by Age category**								
**2-4 years (12 eyes)**	22.0 (±0.5)	<0.001	554.0 (±28.0)	0.69	43.4 (±1.1)	0.026	13.7(±3.3)	0.73
**5-8 years (10 eyes)**	22.9 (±0.9)		555.1(±47.5)		42.0 (±1.6)			
**9-13 years (8 eyes)**	23.5 (±0.6)		541.2 (±34.9)		41.8 (±1.6)		14.1 (±3.0)	

AXL - axial length, CCT - central corneal thickness, K – keratometry, IOP - intraocular pressure

** - p-value from ANOVA

**Figure 1:**
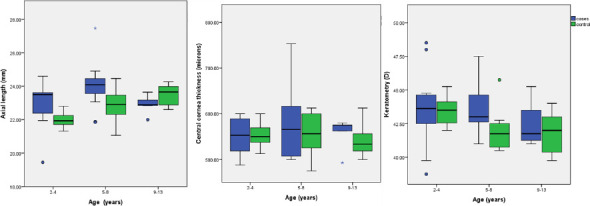
Box plots comparing biometry in normal eyes and in eyes with cataract

The comparison between cataract and control groups according to age categories was graphically represented using the box plot (Figure 1). In the group with cataracts, the trend for longer AXL, steeper keratometry and thicker CCT was primarily noted in the 5 – 8-year-old age group, though not statistically significant. The variances in AXL were larger across the 2 to 4-year-old group in the eyes with cataracts. The increasing trend for longer AXL with increasing age was seen in the children without cataracts, and this was statistically significant.

The presence of linear relationships between the ocular biometric parameters was explored using Pearson's correlation coefficient and the relationship between the ocular biometric parameters and age. In the eyes without cataracts, there was a positive correlation between age and AXL and a strong negative correlation between AXL and keratometry (Table 5).

**Table 5:** Correlation between age and preoperative ocular biometric parameters in normal eyes

	Cases		Controls	
**Parameters**	**r**	**p-value**	**r**	**p-value**
**Age and AXL**	-0.99	0.60	0.62	**<0.001**
**Age and K**	-0.26	0.16	-0.33	0.08
**Age and CCT**	0.13	0.52	-0.19	0.31
**AXL and K**	0.04	0.83	-0.75	**<0.001**
**AXL and CCT**	-0.81	0.69	0.13	0.49
**CCT and K**	-0.24	0.23	-0.33	0.08

AXL - Axial length, CCT - Central corneal thickness, K – Keratometry, r - Pearson's correlation coefficient

## Discussion

In this study, comparing preoperative biometry parameters and CCT in eyes with congenital/developmental pediatric cataract and their age-matched controls, we found that eyes with cataracts had longer AXL, steeper keratometry and thicker central corneas preoperatively. We also noted more variability across all the ocular biometry parameters, though not statistically significant. Significantly positive correlations between age and AXL and negative correlations between AXL and keratometry were noted in the healthy eyes of the control group. These findings suggest that eyes with cataracts do not behave like eyes without pediatric cataracts in African eyes.

The mean age of surgery of children with congenital cataracts at 5.36 years suggests delayed presentation. Boys, who were the majority of the participants undergoing cataract surgery, also had surgery at a younger mean age, which could suggest that boys are likely to access cataract surgery earlier than girls. Studies in other African populations have shown that although the age at detection was similar for both boys and girls with congenital cataracts, boys were brought for care at an earlier age than girls, and more boys received surgery.^15^–^19^ Gender inequalities in cataract surgery among children have also been reported in low-income countries.^20^

There was a significantly longer AXL (23.4 ±1.4, range 19.5 - 27.5 mm) in eyes that had cataracts compared with values obtained in the normal eyes (22.7 ±0.9, range: 21.1 - 24.5 mm). The greater variability in AXL of cataractous eyes is demonstrated by the higher standard deviation, almost 1.5 times the standard deviation in normal eyes. In cataract patients, the maximum AXL value was about 3mm longer than the average values expected for normal adult eyes. This finding supports the reports that pediatric cataracts have been found to disrupt the normal progression of AXL development.^4^ Nihalani et al. noted this difference between cataractous eyes and normal eyes.^12^ and Trivedi et al.^7^ who postulated the reason for this disparity. They proposed that a balance between amblyopia and genetic factors is responsible for the AXL difference between cataractous and normal eyes. In eyes with cataracts, form-deprivation and/or deprivational amblyopia is the stimulus leading to a longer AXL.^7^,^12^

The mean preoperative AXL values in this study compare with those of studies in cataractous eyes in a similar age group using contact biometry in Brazil (23.9mm).^21^ Shorter values were reported in China (22.82mm),^22^ USA (22.3mm),^8^ Netherlands (22.4mm), 23 Nepal (21.94 mm),^24^ and Ireland (21.7mm).^25^ Mean AXL values in normal children in our study are longer than values reported in China (22.45mm), 22 Turkey (22.02mm),^26^ and Turkey (21.99mm).^27^ We hypothesise that this difference could be a result of racial variations.

In both groups in this study, slightly higher AXL values were found in males and children with bilateral cataracts, although this did not reach statistical significance. Significantly longer AXL values have been reported in male children with cataracts.^7^,^8^,^22^,^24^,^26^ Capozzi et al.^28^ found no difference in AXL according to sex. The much younger mean age in the latter study could explain the variation seen. Trivedi et al.^7^ reported longer mean AXL in bilateral cataracts than unilateral cataracts in children younger than 60 months of age, but a shorter AXL in bilateral cataracts than in the eyes with a unilateral cataract in those patients older than 60 months of age. Capozzi et al^28^ found a shorter AXL in children with bilateral congenital cataracts in a study cohort aged 42 months and less. This suggests that the impact of laterality on AXL measurement could be age-dependent. Trivedi et al.^7^ postulated that the form-deprivation and/or derivational amblyopia as stimuli leading to a longer AXL may not be obvious in the first few months of life.^7^ The reason for the sex-linked differences is not clearly stated in the literature.

The overall preoperative mean values for keratometry obtained in the present study were steeper in the cataractous eyes (43.4 ±2.3 D) than in the control group (42.5 ±1.5 D), though not of statistical significance. Steeper corneas in cataractous eyes compared to normal eyes has been found by other researchers.^11^,^28^ The mean keratometry in both arms of the present study is comparable with values reported in China in eyes with cataracts (43.4 D) and without cataracts (42.9 D).^29^ Other researchers reported steeper K values in eyes with cataracts: 44.12D (mean age 7.7 years) in Nepal,23 44.4D (mean age 6.4 years) in Ireland, and 24 and 45.4D (median age 2.25 years) in the USA.^11^ The differences in mean values obtained may be related to the difference in age of the study populations. In much younger children, African-American eyes have been found to have a flatter mean K value compared to Caucasians.^11^

Although slightly steeper keratometry values were found in females and children with bilateral cataracts in the current study, these findings did not reach statistical significance. Steeper corneas have been reported in females and in eyes with bilateral cataracts.^11^,^24^,^29^ The reasons for these findings are not clear in the currently available literature.

Central corneal thickness values preoperatively were just slightly thicker and more variable in the cataract group (563.6 ±59.7 µm) compared to controls (551.0 ±36.4 µm) however, this difference was also not statistically significant. Similar findings obtained with ultrasonic pachymeter in eyes with cataracts have been reported in China (562.98µm),^29^ USA(564µm),^30^ and Brazil (556.24µm).^31^ Also, similar to our findings, thinner pachymetry values have been reported in normal eyes compared to their cataractous counterparts in China (540.29µm),^29^ in Iran (546µm),^32^ in Turkey (559 µm)^27^ and in Turkey (556µm).^26^ Contrary findings of slightly thicker corneas were reported by Faramarzi et al.^33^ in the control group compared to the group with cataracts, although it was also not statistically significant. Their study cohort's younger mean age of 3 years could account for this difference. It is hypothesised that the thicker CCT in the eyes with pediatric cataracts may be due to the delayed development and maturation of the cornea.^29^ Thinner CCT has also been documented in children of African descent when compared to Caucasians.^34^ However, this difference does not appear immediately obvious when directly comparing our studies with the studies as mentioned earlier. In the present study, there were slightly higher values in children with bilateral cataracts and in males; however, this sex difference was only statistically significant in the control group (Lin et al.^29^ We also found thicker pachymetry in eyes with unilateral cataracts and in boys with cataracts compared to girls. The reason for this difference by gender is unknown.

In the group with cataracts, this study did not reveal any inter-relationship between the ocular biometric parameters, nor did we find any correlation between the age at surgery and any of the ocular biometric parameters. This contrasts a study in the USA that showed a strong positive correlation between age at surgery and preoperative AXL.^35^ Their cohort of similar age groups included a significant percentage of patients with traumatic cataracts. The aetiology of traumatic cataracts could suggest that these eyes had normal growth patterns before the insult leading to the cataract. In the control group in this study, a strong negative and significant correlation was found for AXL and K in normal eyes, likely because both parameters exhibited a linear but inverse relationship with age. This relationship in normal eyes is expected in emmetropisation during normal ocular development.

Comparing the preoperative values in normal children and those with cataracts will help our understanding of the association or effect of cataracts on ocular biometrics. A clinical implication of our findings is the choice of IOL power in pediatric cataract surgery. Without keratometry readings for children with cataracts, using normative data for age and sex may be erroneous and lead to undesired postsurgical refraction. This is of import in our environment for two reasons: younger children may not cooperate while being examined with the stationary autorefractor, and secondly, the handheld keratometer is not readily available in most centres in Nigeria. Given that eyes with cataracts have longer AXL, likely due to deprivation amblyopia, we would suggest that this be considered while choosing algorithms for under-correction as a large residual hyperopic refractive error may also be amblyogenic. Research suggests that there may be a need to modify current guidelines for postoperative target refractions to improve long-term refractive outcomes.^36^ Furthermore, longer AXL values obtained during preoperative assessment may assist caregivers in counselling on the expected visual outcomes. Eyes with congenital/developmental cataracts were found to have preoperative IOP values somewhat comparable to those in normal pediatric eyes, as seen in the control group. Identification of elevated IOP during follow-up care of these patients is a central risk factor in the diagnosis and management of glaucoma.

From this study, we recommend that biometric measurements be required for all children going for cataract surgery, as ocular biometric parameters are different in eyes with cataracts compared to normal ones. When using reference values, clinicians should endeavour to use racial-specific values. We also recommend further research with a larger sample size to test the association of other factors that may influence biometry and to create a model to predict ocular biometric parameters in indigenous African eyes.

This study's small sample size was inadequate for multivariate analysis among some sociodemographic and clinical variables. Enrolling two eyes in some children with bilateral cataracts may have introduced a form of bias, as the findings in the second eye may not be independent of the findings in the other eye since both eyes belong to the same person, and there is usually a correlation between the 2 eyes. The correlation of those patients' biometry values could influence the overall analysis.

## Conclusion

There was no statistically significant difference in the mean preoperative central corneal thickness and keratometry values in pediatric cataractous eyes when compared with normal eyes of age-matched controls except for the axial length. There was no significant correlation between age, and all analyzed ocular biometric parameters in children with cataract. The results of this study contribute to the literature, providing data on ocular biometric parameters in indigenous African eyes.
